# Collaboration between dentists and general practitioners in primary care clusters in Hungary

**DOI:** 10.3389/fmed.2026.1879525

**Published:** 2026-07-28

**Authors:** Ferenc Maráczi, Attila Virág, Gergő Túri, Péter Pikó, Csilla Kaposvári, Rita Teller, Csaba László Dózsa, István Vingender

**Affiliations:** 1 Doctoral School of Health Sciences, Faculty of Health Sciences, Semmelweis University, Budapest, Hungary; 2 Faculty of Humanities and Social Sciences, Pázmány Péter Catholic University, Budapest, Hungary; 3 Epidemiology and Surveillance Centre, Semmelweis University, Budapest, Hungary; 4 Synthesis Health Research Foundation, Budapest, Hungary; 5 Department of Public Health and Epidemiology, Faculty of Medicine University of Debrecen, Debrecen, Hungary; 6 Department of Public Health, Semmelweis University, Budapest, Hungary; 7 Faculty of Humanities, Eötvös Loránd University, Budapest, Hungary; 8 Faculty of Health and Sport Sciences, University of Győr, Győr, Hungary

**Keywords:** cluster, collaboration, dentist, financing, general practitioner, Hungary, network, primary care

## Abstract

**Background:**

A substantial challenge in Hungary’s publicly funded primary care system is the rising proportion of unfilled general practitioner and dentist practices over the past several years, which negatively impacts the public’s access to primary care services. The practice cluster model introduced in 2021 aims to improve public access and strengthen collaboration among primary care providers, with a particular focus on interprofessional relationships between general practitioners and dentists.

**Methodology:**

As part of the study, we conducted semi-structured interviews with 36 primary care professionals (18 general practitioners and 18 dentists) between January and March 2026. Sampling was targeted to ensure diversity across geography, demographics, and practice type. We analyzed the data using Mayring’s content analysis method, organized into thematic categories.

**Results:**

The primary motivation for joining primary care clusters was financial incentives, while the development of professional collaboration played a secondary role. The intensity of collaboration between general practitioners and dentists varies; nearly half of the interviewees reported minimal contact. The main areas of collaboration include oral diseases, diabetes, cardiovascular conditions, and medication consultations. Cooperation is most often hindered by heavy workloads, communication gaps, and regulatory and financial constraints.

**Conclusion:**

Primary care clusters offer opportunities for developing interprofessional collaboration; however, systemic barriers limit the realization of this potential. To achieve more effective collaboration, it is necessary to improve communication, establish professional protocols, and reform the financial, regulatory, and infrastructural frameworks.

## 1 Introduction

Primary care systems worldwide increasingly emphasize multidisciplinary collaboration to improve population health outcomes, particularly in the management of chronic diseases. International evidence shows that oral health is closely linked to major non-communicable conditions (including diabetes, cardiovascular disease, and chronic respiratory illness) making cooperation between general practitioners (GPs) and dentists a critical component of integrated primary care (1–4). Several countries, such as the United Kingdom, the Netherlands, Germany, and Italy, have introduced models that strengthen GP–dentist collaboration through shared care pathways, co-located services, and structured communication mechanisms. Despite these developments, interprofessional integration between medical and dental services remains limited in many health systems.

The Hungarian healthcare system is based on a unified social insurance model, which is financed by the National Health Insurance Fund (NHIF) (5–7). Primary care services are generally available free of charge to insured individuals through Hungary’s publicly financed network of general practitioners (GP) and primary care dentists, although a substantial private healthcare sector also operates in the country, particularly in the field of dental care. At the same time, the increasing proportion of vacant general practitioner and dental practices within the publicly financed system has negatively affected access to primary care services for several years, representing a significant health policy challenge (8, 9). Several studies have identified as a substantial challenge the lower accessibility of certain preventive services among primary care providers in Hungary compared to international practices (10, 11).

In recent decades, many developed countries have established clusters, group practices, and integrated care providers based on professional and economic collaboration among various primary care providers, with the aim of improving the public’s access to community-based health care and fostering interdisciplinary collaboration (1–4, 12–14). Following the example of European and North American countries, Hungarian health policy experts have focused on improving primary care since the mid-2010s. As part of this effort, they have established several pilot programs to explore opportunities for collaboration between GPs and other primary care providers (10, 15–17), thereby strengthening primary care as a key pillar of modern healthcare systems.

The GP cluster model introduced in 2021 [Government Decree No. 53/2021. (II. 9.) Government Decree] defines several forms of cooperation: the united cluster (multiple practices under the same provider), the integrated cluster (practices that retain their independence while operating under a jointly established provider), and the GP consortium (18). Within a single district, a group of at least 5 and no more than 10 family practice services may form a GP cluster, and for every 5 GP practices, one primary care dental practice may also be included. Participating practices are required to provide at least 20 h of clinic hours per week—including a minimum of 4 h of preventive care—to submit practice-level data, to establish a joint on-call and substitute schedule, and to apply the preventive care methodologies determined by the practice manager. Under the law, GP clusters may also access grant funds for equipment and human resource development, as well as for integrating additional competencies. The explicit goal of this framework is to strengthen prevention, access, and coordinated patient pathways—including the institutionalization of cooperation between dental and primary care services.

According to data from the NHIF and the National Hospital Directorate (NHD), in the social health insurance system there were 2,603 primary care dental practices and 6,339 GP practices in Hungary in 2025. 10% of dental practices (266) and 15% of GP practices (937) were vacant. Following the launch of the primary care cluster model program in 2021, 78 dental clusters were established by 2025, with 31% of all primary care dentists joining them, and 421 GP clusters were established, with 47% of all GPs joining them (8, 9). One-third of all GP clusters included a dental practice, meaning that these clusters already have practical experience with interprofessional collaboration.

While the model represents a major structural reform, little is known about how collaboration between GPs and dentists actually develops within these clusters, what barriers and facilitators shape their interactions, and whether the model can realistically support integrated primary care.

Existing international studies have examined GP–dentist communication, referral patterns, and interprofessional barriers, but no research has explored these issues within the context of Hungary’s newly implemented cluster system. Understanding how collaboration unfolds in practice is essential for informing future policy decisions, improving the functioning of primary care teams, and contributing to global discussions on integrating oral health into primary care.

The aim of this qualitative study was to explore the experiences, challenges, and opportunities of collaboration between general practitioners and dentists working in Hungary’s primary care clusters, with a focus on identifying system-level and implementation level barriers and generating actionable insights for strengthening integrated primary care both nationally and internationally.

## 2 Methods

### 2.1 Research design

We conducted this research using semi-structured individual interviews to explore collaboration and experiences of dentists and GPs working in primary care, opportunities to improve interprofessional collaboration, and the experiences of primary care practice communities. In designing the study, we endeavored to ensure compliance with the COREQ guidelines for reporting qualitative research, which are detailed in Appendix 1 (19). As a condition of participation, all dentists and GPs provided informed consent in advance, and no personally identifiable data were recorded during the research.

### 2.2 Participants and recruitment

We aimed to recruit at least 18 dentists and 18 GPs working in publicly financed primary care clusters. To ensure maximum variation and broad representation, we predefined sampling quotas along five dimensions: The aim of the study was to interview at least 18 dentists and 18 GPs as follows:

(a) Age: at least two participants from each predefined 10-year age group (30–39, 40–49, 50–59, 60–69, 70–79 years);
(b) Practice location: at least eight dentists and eight GPs from rural areas, eight dentists and eight GPs from urban areas;
(c) Practice type: at least eight dentists and eight GPs from adult practices and at least eight dentists and eight GPs from mixed (adult and pediatric) practices;
(d) Cluster membership: only one interviewee was recruited from each GP cluster to avoid clustering of perspectives;
(e) Geographic coverage: at least two primary care dentists and two GPs from each of Hungary’s statistical regions.

Potential participants were identified using the NHIF’s publicly available online database of primary care providers, which enabled targeted selection of dentists and GPs across different regions, practice types, and settlement characteristics. Selected professionals were contacted via their workplace email addresses and invited to participate in the study. Those who expressed interest completed a short online questionnaire collecting basic sociodemographic and practice characteristics (e.g., age group, region, practice type, urban/rural), as well as their availability for an interview. Based on these responses, we scheduled interviews electronically to fulfill the predefined sampling quotas.

Eligibility criteria required that participants (1) were practicing as primary care dentists or GPs in Hungary, (2) were members of a primary care GP cluster at the time of the study, (3) were willing to share their experiences regarding the functioning of clusters and interprofessional collaboration, and (4) provided prior written informed consent.

### 2.3 Data collection and processing

In line with the research objectives, we developed a semi-structured interview guide by adapting and further developing the interview framework published in the study by Sippli and colleagues, tailoring it to the Hungarian context and incorporating the topic of GP clusters into the investigated field (20). The topics were as follows: (a) experiences with establishing primary care clusters; (b) areas and experiences of professional collaboration between general practitioners and dentists; (c) opportunities for developing interprofessional collaboration. The interview questions were not shared with participants in advance to avoid priming effects. The guide was pilot-tested with one dentist and one GP to assess clarity and relevance, and minor wording adjustments were made accordingly.

The interviews were conducted between January and March 2026 by six trained researchers (FM, RT, CD, CK, AV, and GT), all of whom had prior experience conducting qualitative interviews in health services research. All interviews were recorded via Zoom teleconference, or by smartphone. The interviews were transcribed verbatim from the audio recordings into Microsoft Word by FM, CK and GT, and the transcripts were checked by FM for consistency. Participants did not review transcripts, as the study focused on system-level experiences rather than personal narratives. The semi-structured interview guide is included in Appendix 2.

While conducting the interviews, we continuously monitored data saturation, the point at which further data collection is unlikely to yield new, meaningful information. According to the literature, this is typically reached after 7–18 interviews. In this study, in order to obtain a comprehensive picture of the relevant topics for both professions, we assessed data saturation separately among GPs and dentists. In the assessment, we applied the approach described by Greg Guest and colleagues, using a 5% threshold for new information (21). A total of 36 interviews were conducted (18 per profession), which were sufficient to achieve thematic saturation and to explore a broad spectrum of relevant content.

During the analysis, we followed the principles of data triangulation proposed by Carter et al. to enhance data reliability (22). Six researchers with diverse professional backgrounds conducted the interviews, while two researchers were responsible for coding the transcripts and developing the coding framework. We included general practitioners and dentists with diverse characteristics in the study to ensure sample diversity.

### 2.4 Analysis

We conducted the content analysis using Philipp Mayring’s methodological approach (23).

First, we summarized participants’ basic characteristics using descriptive statistics (age group, sex, practice type, cluster membership, geographic region, and settlement type) to contextualize the qualitative findings. The analytic process began with an inductive development of the category system. Two researchers (FM and GT) independently reviewed and summarized the first 10 interview transcripts (five GPs and five dentists), identifying recurring content elements and meaning units. Based on these summaries, we generated an initial set of inductive categories.

Following Mayring’s structured content analysis, we defined the coding unit (a coherent meaning-bearing segment, typically a sentence or short paragraph) and the context unit (the full interview transcript). Using these units, we refined the initial categories into a hierarchical system of main categories and subcategories. This category system was iteratively revised as additional transcripts were analyzed, ensuring that new themes were incorporated and that category definitions remained consistent and analytically meaningful.

All transcripts were coded using Atlas.ti 22 software. FM and GT independently coded each transcript in at least two rounds, applying and refining the coding framework. Coding discrepancies were discussed and resolved through consensus, and the evolving category system was reviewed by the full research team to strengthen intersubjective reliability. This iterative team-based process ensured that interpretations were grounded in the data and not influenced by individual researcher bias.

After coding was completed, we synthesized the content of each subcategory into thematic units. These thematic units were then organized to address the study’s three main analytic focus: (1) experiences related to the formation and functioning of GP clusters; (2) characteristic areas and practical experiences of professional collaboration between GP and dental practices; and (3) potential directions for strengthening interprofessional collaboration and the cluster model. This structured analytic approach enabled us to identify system-level mechanisms, barriers, and facilitators relevant to the functioning of primary care clusters.

The detailed coding process generated a larger number of inductive subcategories, which were subsequently grouped into the three overarching analytical themes presented in Table 1.

**Table 1:** Overview of the qualitative coding framework.

Main analytical focus	Main categories	Subcategories
Experiences with GP clusters	Motivation for joining; Operation of GP clusters	Financial incentives; professional development; infrastructure; division of labor
GP–dentist collaboration	Professional relationships; Areas of collaboration	Oral diseases; diabetes; medication-related consultation; preventive care; referral pathways
Future development	Barriers; Facilitators; Improvement opportunities	Communication; education; protocols; financing; digital systems

Sources: Authors.

## 3 Results

### 3.1 Sample description

A total of 36 primary care professionals participated in the study, comprising 18 dentists and 18 GPs. The average interview duration was 47 min. The sample reflected the predefined maximum-variation sampling strategy: participants represented all major age groups, a balanced gender distribution (53% women), both adult and mixed practices, and all regions of Hungary. At least two dentists and two GPs were included from each region, and the sample covered urban, rural service areas. Most participants worked in urban practices, reflecting national patterns of primary care distribution. Table 2 summarizes the demographic and practice characteristics of the interviewed participants.

**Table 2:** Demographic and practice characteristics of interviewed dentists and general practitioners (GP) (*n* = 36).

		Dentist	GP
Age groups (years)	30–39	4	3
	40–49	4	4
	50–59	3	4
	60–69	4	4
	70–79	3	3
Sex	Female	9	10
	Male	9	8
Practice type	Adult	9	8
	Mixed	9	10
Region	Western Transdanubia	2	2
	Southern Transdanubia	2	2
	Central Transdanubia	2	2
	Central Hungary	3	2
	Budapest	3	3
	Southern Great Plain	2	2
	Northern Great Plain	2	2
	Northern Hungary	2	2
Area	Urban	10	9
	Rural	8	9

Sources: Authors.

### 3.2 Experiences with the establishment and operation of primary care GP clusters

#### 3.2.1 Motivations for joining a GP cluster

The majority of interviewees joined GP clusters primarily because of financial incentives, as practices participating in the new operating model received wage subsidies. Some of the interviewees also cited the opportunity to apply for equipment and infrastructure development grants, intended for practices that joined GP clusters, as a motivation; however, they noted that few such grants have been announced since 2021.

*GP10: “Additional funding was the most important factor in joining the cluster, as it translates to hundreds of thousands of forints in monthly revenue for each practice, which is a positive change. However, only a few grants have been announced so far for the development of practice facilities, and there is a need for much larger-scale improvements.”*

In addition, some interviewees cited professional development opportunities and the potential for enhanced collaboration between general practitioners and dentists as sources of motivation. This motivational factor was typically mentioned by younger general practitioners and dentists (age group: 30–39). Among older interviewees, another reason cited was that it is easier for them to coordinate cover shifts within the cluster.

*Dentist 12: “As a new graduate, I’m also motivated by the fact that I can discuss more complex cases with my colleagues in the cluster, which helps me learn and grow. In the cluster, we hold these professional meetings every few months, where we occasionally have the opportunity to review and discuss one or two case studies, which I find very useful.”*

#### 3.2.2 General experiences with the operation of primary care GP clusters

According to the majority of interviewees, GPs and dentists who have joined GP clusters are able to meet the conditions for additional funding, including providing regular data reports, ensuring on-call services, and providing preventive care to the public. The majority of GPs and dentists who have joined GP clusters continue to work on their own, and the division of labor among practices has not yet fully materialized. This is partly because the practices participating in GP clusters are often not located in the same building, but rather in settlements or parts of settlements that are far apart. The interviewees perceived the spatial distance between practices as an obstacle to professional collaboration, and, as a result, GP clusters have a more limited ability to jointly employ other primary care professionals or to use larger, less mobile diagnostic and therapeutic equipment. The division of labor among practices is further hindered by the fact that GPs’ scope of practice and funding algorithms continue to limit a GP’s ability to refer a patient to a colleague within the GP cluster who holds the relevant speciality certification, rather than to a clinic or hospital. As a result, it is currently difficult to ensure, within the framework of GP clusters, that patients can access specific healthcare services as close to their place of residence as possible and at the lowest possible level of progressive care.

*GP 15: “If there are five GPs in a cluster, and each of them has an additional speciality certification—for example, one in pulmonology, another in psychiatry, and a third in internal medicine—they all have the same legal rights. I cannot refer my patients to them because I am not authorized to do so, and these GPs are not authorized to perform the relevant examinations in their practice or to prescribe medications, even though they have the necessary qualifications. And even if they were equipped with the tools necessary to care for a given patient group, the payer would not reimburse the cost of these tests and therapies.”*

*GP 6: “I hold several speciality certifications, and it often happens that I have to refer my own patient to another clinic or hospital, solely so that I can prescribe the necessary medications with a different stamp.”*

Interviewees who joined a GP cluster where a substantial proportion of the practices are located within a single municipality and a single institution typically reported more active collaboration and found it easier to share diagnostic and therapeutic tools as well as additional staff. Several interviewees also emphasized that they had already placed strong emphasis on prevention before joining the GP cluster, so no significant change in the types of services they provide can be identified.

### 3.3 Fields and experiences of professional collaboration between GPs and dentists

#### 3.3.1 Professional relationships between GPs and dentists

The interviewees had differing views on the relationships between the two professions. According to nearly half of GPs and dentists, minimal contact and collaboration can be identified within general practices and dental practices operating within the same GP cluster, as well as between practices operating outside the cluster. Nearly a quarter of the GPs and dentists interviewed reported that a more active professional relationship between dentists and GPs has begun to develop within the cluster. Another quarter of the interviewees reported more active cooperation and relationships with GPs or dentist practices operating outside the GP cluster. A more common statement among the GP interviewees was that they were less familiar with the dentists operating in their service area. They cited two reasons for this: first, they felt there was greater fluctuation among dentists, and second, the service areas of general practitioners and dentists often did not overlap.

*GP 2: “Most of my patients are not treated by the same dentist, but by publicly funded or private dentists practicing in different parts of the city; therefore, it is not possible to speak of a continuous, long-term collaboration with a specific colleague. Rather, there are connections regarding specific patients or conditions for the duration of one or two visits.”*

GPs and dentists typically communicate by phone, email, or via a referral form given to the patient. Some GPs and dentists reported that they occasionally receive verbal messages from representatives of the other profession through their patients, which they consider unacceptable. However, according to the interviewees, some patients may simply be trying to obtain prescription medication this way rather than visiting a dentist or GP relevant to their condition.

*Dentist 5: “I communicate with the patient’s GP primarily through the medical referral, in which I describe the symptom or problem that leads me to believe further examination or consultation is warranted. I think it’s important to clearly inform my colleague why I’m referring the patient to them; we could lose important information if we rely solely on verbal communication through the patient.”*

#### 3.3.2 Areas of collaboration between GPs and dentists, according to GPs

In order to facilitate interpretation of the findings, Table 3 summarizes the main thematic areas of collaboration identified during the qualitative analysis and illustrates them with the specific clinical conditions and situations reported by the interviewees. The detailed findings for each theme are presented in the subsequent sections.

**Table 3:** Clinical situations identified by interviewees grouped into broader collaboration themes.

Collaboration theme	Examples identified by participants
Chronic disease management	Diabetes, cardiovascular disease, hypertension, liver disease, reflux, osteoporosis
Medication-related consultation	Anticoagulants, bisphosphonate therapy, allergy assessment, antibiotic consultation
Oral disease management	Periodontitis, oral infections, abscesses, cysts, gingival bleeding, oral inflammation
Preventive care	Oral cancer screening, smoking cessation, dental screening, oral examinations
Functional disorders	Denture-related problems, chewing disorders, swallowing problems, salivation disorders
Referral for systemic conditions	Weight loss, neurological disorders, tinnitus, mental health disorders, facial swelling, lymph node enlargement

Sources: Authors.

##### 3.3.2.1 Health conditions and symptoms for which GPs refer their patients to a dentist

Most of the GPs interviewed recommended that patients visit a dentist if they have neglected teeth, toothaches, broken teeth, oral inflammation of unknown origin, recurrent oral infections, chewing and swallowing problems, mouth sores, abscess-like lesions, periapical cysts, or bleeding gums. The majority of GPs also identified dental screening as an area of collaboration, which is a relevant diagnostic test for investigating the underlying causes of hair loss, joint problems, and cardiovascular issues. Furthermore, dental focal infection elimination may be necessary prior to certain surgical procedures, such as heart valve replacements. In addition to all this, the interviewees frequently mentioned referrals for oral examinations when suspicious lesions are identified on the lips or in the oral cavity, or when swelling is detected in the facial or cervical lymph nodes.

*GP 3: “I usually remind my patients who smoke heavily of the importance of oral examinations, and I recommend that they visit their dentist from time to time for this purpose. Furthermore, dental decontamination is mandatory prior to certain surgical procedures, and as a GP, I usually refer patients for this.”*

*GP 6: “In Hungary, the oral health of the population is generally very poor; I believe that the majority of patients who come to my practice could benefit from seeing a dentist.”*

Some GPs also mentioned that they consider referral to a dentist justified even if the patient has a salivation problem, or if the patient has dentures but does not use them or uses them improperly, since failure to chew can cause digestive problems. Several interviewees also mentioned that a dental consultation is necessary before taking bisphosphonate-containing medication used to treat osteoporosis, because one possible side effect of the medication is osteonecrosis of the jaw, and the risk of this must be assessed before starting therapy.

##### 3.3.2.2 Health conditions and symptoms that, according to GPs, dentists can recognize and subsequently refer the patient to their general practitioner

The condition mostly mentioned by GPs was diabetes, which can manifest in symptoms detectable by dentists, such as bad breath, rapidly deteriorating dental health, gum recession, and loose teeth. The interviewees also cited liver disease and reflux as potential causes of bad breath. The condition of the teeth can also be affected by acid secretion disorders and reflux, which warrant referral to a primary care physician. Another area of collaboration frequently mentioned by most general practitioners is tooth extraction for patients taking blood-thinning medications and pre-operative consultation for oral surgery, during which the dentist coordinates the patient’s medication regimen with the general practitioner.

*GP 1: “*Var*ious types of bad breath can stem from conditions ranging from diabetes to reflux, and even liver disease. In the case of diabetes, another telltale sign is the progressive deterioration of the oral cavity. In recent years, there have been a few instances in my practice in which a dentist or another specialist referred a patient to me for further evaluation due to these symptoms.”*

Several interviewees also mentioned that patients with a history of substantial tobacco exposure who visit a dental clinic can be referred to smoking cessation programs offered by their GPs. According to some GPs, certain mental health conditions can also cause oral health problems (for example, teeth grinding due to anxiety or jaw clenching can worsen the condition of the teeth, and may indirectly cause or exacerbate tinnitus), which require a referral to a general practitioner.

*GP 9: “I can share an interesting example from last year that illustrates the importance of collaboration between GPs, dentists, and other specialists. A young patient suffering from tinnitus came to my practice. Since ENT, cardiological, and neurological examinations could not identify the underlying cause, I thought it appropriate to refer the patient for psychological and dental evaluation as well. Subsequently, it was determined that due to workplace stress and anxiety, the patient regularly grinds their teeth at night and clenches their jaw. By using the relaxation techniques provided by the psychologist and the nighttime mouthguard recommended by the dentist, we were able to significantly reduce the symptoms.”*

Some primary GPs have also emphasized that the complications and chronic inflammatory effects of periodontitis can be linked to cardiovascular diseases and, if the infection spreads locally, can also cause periostitis; therefore, a consultation with a GP may be warranted. A referral to a GP may be required if a patient presenting at a dentist’s office has chewing difficulties and, due to significant weight loss, requires nutritional supplements, which the general practitioner can prescribe for them. Some interviewees also emphasized that toothaches and headaches may be caused not only by dental issues but also by neurological conditions (e.g., neuralgia), and in the case of headaches, hypertension may also play a role, which may warrant further investigation.

A few interviewees also mentioned that dentists can identify suspicious skin lesions on patients’ faces and necks, as well as suspicious ophthalmic problems (such as strabismus in young children), and that chronic tonsillitis and benign tumors may also require consultation with a GP.

##### 3.3.2.3 Areas of collaboration between GPs and dentists, according to dentists health conditions and symptoms for which dentists refer their patients to general practitioners

Dentists most frequently mentioned symptoms related to diabetes; if they identify these during dental treatment, they refer their patients to a GP. According to the interviewees, symptoms and conditions indicative of diabetes or untreated diabetes include bad breath, periodontal disease, and recurrent, ulcerated mucosal lesions. Several interviewees also mentioned that, since dentists are not authorized to prescribe sick leave, they must refer patients who require sick leave following dental treatment to their GP.

*Dentist 15: “Diabetes requires a completely different approach to dental care. Diabetes—and especially untreated diabetes—can manifest itself in bad breath and in the condition of the oral cavity and gums.”*

According to the majority of the dentist interviewees, it is sometimes necessary to contact the patient’s GP before a tooth extraction or minor oral surgery if the patient is taking anticoagulants or has a complex medication regimen that could affect treatment. Consultation with the family physician may also be necessary if the patient’s drug sensitivities or allergies are unknown. Some dentists mentioned that consulting the patient’s own GP can be replaced if they can access the patient’s medical history and regularly taken medications through Hungary’s Electronic Health Service Space (EESZT), or if there is a GP within the cluster with whom they can consult on the case. In addition, some dentists mentioned that, regarding the use of antibiotics, they consider consultation with the patient’s dentist warranted.

*Dentist 3: “For patients taking anticoagulants or those with very complex medication regimens, I usually consult with the patient’s primary care physician before a tooth extraction. If they are unavailable, I can seek the opinion of another primary care physician colleague working in our cluster.”*

Some interviewees also recommended that patients consult a GP if they experience fluctuating blood pressure during dental treatment or if they are diagnosed with periodontitis. A dentist further highlighted that on one occasion, following the administration of a local anesthetic, the patient developed unusually severe soft tissue hemorrhage and facial swelling. In the dentist’s view, this was a suspicious sign suggestive of acute leukemia, which was subsequently confirmed by diagnostic examinations after the dental treatment.

*Dentist 12: “If a patient experiences significant fluctuations in blood pressure during treatment—and there are visible signs of this—I usually point out that, although I am not their GP, it is worth seeing one, because an untreated chronic condition is not the secret to a long life.”*

##### 3.3.2.4 Health conditions and symptoms that dentists believe GPs can recognize and subsequently refer the patient to a dentist

Most of the dentist interviewees agreed that it would be beneficial if GPs recommended regular dental check-ups and follow-ups to a significant portion of their patients. According to dentists, GPs can identify when patients visiting their offices have neglected oral health, poor dentition, or gum problems. Many dentists emphasized that regular dental scaling is advisable, and GPs can also bring this to their patients’ attention.

*Dentist 2: “Many people are unaware that dental calculus can contribute to the development of periodontal disease. Dental calculus removal is not necessarily an invasive procedure, and many patients do not know that they are eligible for this treatment every 6 months under the social health insurance system.”*

Several interviewees also emphasized that while GPs do not have all the tools or comprehensive training to perform oral examinations, they can refer patients who are regular smokers, have neglected oral health, or exhibit suspicious oral lesions to dentists.

*Dentist 14: “If a patient presents to a GP with simple cold symptoms and the doctor looks inside their mouth, they can see the condition of the patient’s oral cavity. If they observe any extreme abnormalities—which could be a precancerous condition, any other drastic abnormality, or facial swelling—they can refer the patient to us.”*

Some dentists also mentioned that GPs may refer patients to them if they notice a coated tongue or identify fungal infections on the oral mucosa. Some of the interviewees also mentioned potential problems and symptoms associated with denture use, including mechanical injuries resulting from improper denture use and oral ulcers. In addition, it would be advisable for GPs to emphasize the importance of dental prosthetics to patients with missing teeth.

#### 3.3.3 Opportunities for interprofessional collaboration and the development of GP clusters

##### 3.3.3.1 Factors supporting and hindering collaboration

According to the majority of the GPs and dentists interviewed, the main factors hindering collaboration are doctors’ heavy workloads and a lack of appropriate communication methods and skills. Several interviewees also identified as a barrier the fact that the service areas of GPs and dentists do not overlap, as well as that a substantial proportion of the population uses private dental services instead of publicly funded dental care. Some interviewees also noted that in certain cases, the contact information for primary care providers and specialists relevant to patient care is less accessible, which can hinder their ability to quickly consult with colleagues in related fields via phone or email regarding a specific case.

*GP 13: “The problem is that proper communication methods have not been established between GPs and dentists. There are still colleagues who send messages through the patient asking me to prescribe a medication for them or refer them for an examination somewhere. The proper procedure would be for them to write a medical referral stating the patient’s complaint, the appropriate medication, or the indication for a diagnostic test.”*

According to the majority of interviewees, factors that support collaboration include existing positive personal relationships and the presence of representatives from multiple professions within the same building, which facilitates quick, personal communication regarding individual cases. Some of the interviewees also emphasized that they consider the professional dedication and openness of GPs and dentists toward related fields to be a factor supporting interprofessional collaboration, thanks to which they may be able to improve their patients’ health.

*Dentist 11: “In my opinion, a key factor in collaboration is the colleague’s personality, which also enables effective professional collaboration.”*

*GP 4: “Having multiple professions at a single location greatly facilitates close collaboration because we see each other every day, and I only have to take a few steps if I want to consult with another colleague about a case.”*

##### 3.3.3.2 Opportunities for developing interprofessional collaboration

According to the majority of interviewees, the development of interprofessional collaboration would be considerably enhanced by joint professional medical education programs where participants could learn about related fields—whether through case studies or by building personal connections. According to GPs and dentists, they have rarely, if ever, attended professional medical education courses where representatives of both or more professions were present, and they have rarely, if ever, encountered a presentation that explored opportunities for collaboration between the two professions.

*Dentist 18: “I would find it useful to have continuing medical education focused on the points of connection and opportunities for collaboration between GPs and dentists, and to get to know my colleagues who are GPs and dentists working in my town or county. Effective collaboration techniques could be disseminated through the sharing of existing best practices.”*

A majority of the interviewees also considered it useful to establish professional protocols that provide guidance to GPs and dentists on when collaboration with related professions may be warranted for certain symptoms or health issues. Some of the interviewees also emphasized that standardizing patient pathways and supporting the organization of patient pathways would be a particularly important area for development in interprofessional collaboration.

*GP 10: “Cooperation with related professions could function effectively and consistently if there were a protocol specifying how and to which specialist a patient should be referred for a given problem. In the current system, this is sometimes less transparent. Such protocols could even be developed for specific patient groups where interprofessional collaboration may be necessary.”*

Some of the interviewees also emphasized that cooperation would be facilitated by the creation of communication platforms that would provide information about programs in related professions, as well as the telephone and email contact information for primary care providers, where healthcare professionals could communicate directly and discuss with one another regarding specific cases.

*GP 9: “It would be good to be notified when dentists launch a preventive program to which I can refer my patients. It would also be helpful if there were a platform accessible to a limited group of users that listed healthcare providers’ workplace contact information; this would greatly facilitate patient care coordination and professional consultation.”*

##### 3.3.3.3 Opportunities for developing GP clusters

According to the majority of interviewees, it would be advisable to involve additional professionals in the operation of GP clusters. GPs most frequently mentioned dietitians, physical therapists, mental health professionals, and other administrative and assistant roles, while dentists most often cited dental hygienists, other administrative and assistant roles, and mental health professionals. However, the majority of interviewees agreed that current financing, healthcare organization, and other legal regulations, as well as infrastructural factors, do not allow for the expansion of practice communities’ capacities. In addition to these, securing additional financial resources was also a frequently raised need among the interviewees. Some interviewees also emphasized that they consider the development of clusters’ human resource capacity acceptable only if the currently scattered site structure is eliminated and integrated into regional centers, where up to 10–15 specialities could be accommodated within a single building. Regarding infrastructure developments, the need for improvements in transportation planning was also highlighted, which is a particularly relevant factor for practice communities operating in rural, geographically dispersed settlement structures, as it affects the population’s access to healthcare services.

*GP 18: “If I, or our GP cluster, wanted to hire a dietitian or a physical therapist, we would need to provide them with a space and the equipment they need to work. Even if we had the space and equipment, the issue of referral authority would arise again, since only a rheumatologist—not a general practitioner—can refer patients for physical therapy. So the area of care organization that influences patients’ care pathways would also need to be improved. And we have not even mentioned the funding rules, which currently do not provide for the financing of additional professional capacity.”*

*GP 4: “It is not sustainable for a GP practice to continuously operate at a loss, or for the specialists employed in the GP cluster to work for low wages. More resources need to be allocated within the system for these tasks.”*

Both the dentist and GP interviewees raised the need for equipment upgrades for diagnostic and therapeutic purposes. However, the topic of equipment purchased jointly for the GP cluster divided the interviewees, as several participants emphasized that there are currently no clear guidelines on how to jointly use, operate, and maintain the equipment, and that moving and storing larger pieces of equipment between locations can also pose problems.

*Dentist 6: “For now, I do not foresee the operation of a jointly acquired diagnostic or therapeutic device. Legally, I believe this could be registered under one practice’s name and then made available to other practices through a loan-for-use agreement; however, it would need to be clarified how the costs and responsibilities for operation and maintenance would be divided among the practices.”*

The majority of the GP and dentist interviewees also emphasized that the Hungarian population’s health literacy and health culture lag substantially behind the desired level, and that considerable resources should be allocated to improve this. The area most frequently mentioned by the interviewees was the development of literacy regarding healthy lifestyles, prevention, medication use and adherence, the use of the healthcare system, and patient pathways.

*GP 3: “An important step in healthcare reform is patient education, so that patients know not to go to the hospital emergency room at 10 p.m. with a fungal nail infection they have had for three months, or so that they know what to do if a colleague faints at work.”*

Some of the GPs interviewed also emphasized that the indicators used to evaluate their performance are in need of reform, as they currently include several elements whose fulfillment depends not solely on the effectiveness of the GP’s work, but also in part on patient behavior, as well as other social and health system factors.

*GP 10: “It is pointless for the performance evaluation system to require the highest possible flu vaccination rate among those over 60 if I only receive 50 doses of vaccine for my practice of 2,000 patients. It is also a requirement that the proportion of patients with high blood lipids in the practice be as low as possible, but if patients do not follow the recommendations provided in lifestyle counseling, or if they cannot afford to purchase cholesterol-lowering medication, or if it is not available at the pharmacy, this negatively impacts the GP’s performance evaluation.”*

## 4 Discussion

Although previous Hungarian studies have examined the establishment and operation of dental clusters, the present study differs in two important respects (8, 24). First, it focuses on the recently introduced GP cluster model rather than the dental cluster model. Second, instead of examining the functioning of dental clusters alone, it specifically explores the experiences of collaboration between general practitioners and dentists working within the GP cluster framework. This provides the first qualitative evidence on how interprofessional collaboration develops within Hungary’s new primary care organizational model.

The results of our study are consistent with those of a previously published study conducted exclusively among dental clusters in Hungary, which found that joining primary care dental clusters was primarily driven by financial motivations (24). Our findings also indicate that professional development and the opportunity to enhance collaboration emerged as motivations, which is consistent with the results of the previous analysis. A new finding of our research is that the division of labor within GP clusters is hindered by numerous factors, notably the more limited scope of authority of primary care providers and the financing system. However, the results of our current study confirmed the earlier findings of Sztrilich and colleagues, namely that more active collaboration developed in clusters where a substantial proportion of the participating practices operate at a single location (24).

According to our findings, nearly half of GPs and dentists reported minimal interprofessional collaboration within their GP cluster or with other professionals working in their service area. However, the other half of GPs and dentists reported more active professional collaboration. Sippli and colleagues reported results partially consistent with ours; they found varying degrees of collaboration among general practitioners and dentists in Germany, but their study indicates that dentists have a greater need for professional collaboration than GPs (20).

According to our findings, GPs and dentists most frequently identify areas for collaboration with representatives of related professions in the treatment and prevention of oral diseases, diabetes, and cardiovascular diseases, as well as in eliminating dental focal infections and using certain medications. Our findings are consistent with research conducted in numerous developed countries, which also identified the treatment and prevention of diabetes and oral diseases, smoking prevention, healthy nutrition, and medication consultations prior to certain dental procedures (anticoagulants, bisphosphonate-containing medications) as the most important areas of collaboration (20, 25, 26). These studies also highlight that interprofessional collaboration not only facilitates disease prevention, early detection, and treatment, but also offers opportunities to improve the coordination of patient care.

According to our findings, collaboration between GPs and dentists is most often hindered by physicians’ heavy workloads and a lack of appropriate communication methods and channels. The authors of two systematic reviews relevant to this topic also highlighted inadequate communication as a factor hindering interprofessional collaboration, but they also identified unequal power dynamics, which were less frequently mentioned by the interviewees in our study (3, 27). According to our research, the factors supporting interprofessional collaboration include positive personal relationships between dentists and GPs, the proximity of their practices, as well as a shared professional commitment and openness to collaboration. Our results are consistent with the findings of systematic reviews, which indicate that shared professional goals and the proximity of providers also facilitate the development of close collaboration and effective division of labor.

Our findings suggest that digital infrastructure plays a dual role in supporting interprofessional collaboration. Several dentists reported that access to patient records, medication histories, and other clinical information through Hungary’s Electronic Health Service Space (EESZT) facilitates safer and more informed clinical decision-making. However, despite the availability of these digital resources, communication between general practitioners and dentists remains largely dependent on telephone calls, e-mails, and referral letters. This indicates that while EESZT provides valuable access to patient information, it does not in itself substitute for structured communication pathways between healthcare professionals. Future developments could therefore focus on digital solutions that facilitate direct professional consultation and coordinated care between primary care providers.

Among the opportunities to improve interprofessional collaboration between GPs and dentists, we identified implementing joint continuing education programs and developing professional protocols. The study by Sztrilich and colleagues yielded results consistent with our research, indicating that dentists would view the development of protocols standardizing professional procedures and collaboration, as well as the implementation of continuing medical education aimed at knowledge sharing, in which, in addition to dental practices, other primary care providers, such as GPs, could also participate (24).

According to our findings, there are numerous opportunities to improve the operation of GP clusters. In line with international trends, Hungarian GPs and dentists would also find it beneficial to involve additional professionals in the operation of clusters, such as dietitians, physical therapists, mental health professionals, and dental hygienists. However, among Hungarian professionals, there has been less discussion of involving other primary care providers in the operation of clusters, such as pharmacists or professionals providing prenatal care, who are, however, part of primary care collaborations in the United Kingdom and the Netherlands (28–30). However, the GPs and dentists surveyed in our study emphasized that involving additional specialists would only be advisable following the necessary infrastructure and equipment upgrades, as well as reforms to jurisdictional authority and funding regulations. Our findings are consistent with international evidence suggesting that structural integration alone does not automatically result in effective interprofessional collaboration. Experiences from countries such as the United Kingdom and the Netherlands indicate that successful collaboration between primary care providers is often supported by shared care pathways, regular multidisciplinary meetings, and well-established communication mechanisms. The results of our study suggest that the Hungarian GP cluster model has primarily succeeded in establishing the structural framework for collaboration, while the organizational and professional mechanisms required for routine interdisciplinary cooperation remain under development.

Based on the interviews, participants perceived that the long-term sustainability of the GP cluster model could be strengthened by ensuring that infrastructure development resources accompany the existing financial incentives. Several interviewees reported that relatively few equipment and infrastructure development programs had been launched since the introduction of the model. These perceptions suggest that participants viewed the current system as placing greater emphasis on workforce financing than on developing the physical, digital, managerial, and organizational capacities needed for multidisciplinary collaboration. While these findings do not demonstrate the effectiveness of specific policy interventions, they highlight areas that participants considered important for the future development of the GP cluster model.

Our research has the following limitations. Firstly, qualitative research is inherently characterized by a certain degree of subjectivity in interpretation; however, in this study, we ensured the inclusion of participants with diverse backgrounds and differing perspectives through deliberate sampling. Data processing and interpretation were conducted in accordance with established methodological guidelines, with the active participation of the entire research team, thereby reinforcing the reliability and validity of the results (23). Secondly, our study covered the first 4 years of the GP clusters’ formation and operation—that is, the experiences of the introductory phase—during a period influenced by several substantial economic and social factors. Nevertheless, the initial operational experiences can already provide valuable lessons at this early stage and contribute to the further development and refinement of the GP cluster model, as well as cooperation between GPs and dentists. A further limitation of our study is that GPs and dentists providing care for children were not included; however, this was partly due to the fact that providers of pediatric dental services are not eligible to join GP clusters.

An additional limitation of our study is that it exclusively explored the perspectives of general practitioners and dentists. Consequently, we were unable to assess whether patients themselves perceived any changes in access to care, care coordination, or service quality following the introduction of the GP cluster model. Future research should therefore incorporate patient perspectives and experiences to provide a more comprehensive evaluation of the model’s impact. Furthermore, participants were recruited through workplace e-mail invitations and an online registration process. Although the maximum variation sampling strategy enabled us to capture experiences from participants representing different age groups, regions, and practice settings, it is possible that the study disproportionately attracted professionals who were more open to discussing GP clusters and interprofessional collaboration. Therefore, the findings should not be interpreted as statistically representative of all Hungarian general practitioners and dentists. Finally, this study employed a cross-sectional design and relied on participants’ retrospective reflections regarding the implementation of GP clusters since 2021. As no qualitative baseline data were collected prior to the introduction of the reform, the findings primarily reflect participants’ perceptions and experiences rather than objectively measured changes over time. Another limitation of our study is that it focused exclusively on dentists and general practitioners participating in Hungary’s publicly financed primary care system. Although this system provides care for a substantial proportion of the population, particularly in general practice, a considerable share of dental care is delivered in the private sector. Consequently, the findings may not fully reflect collaboration patterns, referral practices, and communication mechanisms involving private dental providers.

A promising future research direction involves identifying and evaluating models of collaboration between primary care GP clusters and other key stakeholders, such as health promotion offices, dietitians, physical therapists, and mental health professionals (31). Another important area of research is the potential to adapt telemedicine services and point-of-care diagnostic tests within medical practices (15, 32, 33). It is also essential to establish a monitoring system that enables the ongoing evaluation of the operations of primary care GP clusters, as well as to conduct continuous, systematic assessments of their operations.

## 5 Conclusion

The study highlighted that although the GP cluster model provides a framework for strengthening collaboration between GPs and dentists, in practice, this collaboration remains limited. Joining the cluster was primarily motivated by financial incentives, while professional integration and the development of a genuine division of labor often failed to materialize. The intensity of collaboration is substantially influenced by the practices’ geographical location, the presence of personal relationships, and the quality of communication. Based on the results, key factors for improving interprofessional collaboration include establishing structured communication channels, strengthening informal communication channels, organizing joint professional training, and developing standardized professional protocols. When developing future interprofessional protocols, priority could be given to clinical areas that were frequently identified by participants as requiring collaboration between general practitioners and dentists. These include the management of patients with diabetes and oral health problems, the coordination of care for patients receiving anticoagulant therapy who require dental interventions, referral pathways related to oral cancer screening, and the management of patients with chronic periodontal and cardiovascular conditions. Standardized care pathways in these areas may contribute to more effective collaboration and improved patient outcomes. In addition, making patient pathways more transparent and coordinated is of paramount importance, as this can contribute to more efficient care organization and increased patient safety. The interview findings suggest that future reforms may benefit from considering changes to financing mechanisms, regulatory frameworks, and organizational arrangements that could facilitate multidisciplinary collaboration. In the future, the success of the model may largely depend on the extent to which current structural barriers can be reduced and effective, patient-centered collaboration among primary care providers can be facilitated.

## Data Availability

The datasets presented in this article are not readily available because of the nature of the qualitative study method, and to protect study participants privacy. Requests to access the datasets should be directed to Gergő Túri, turi.gergo@semmelweis.hu.
