# Supplementary material for: Collaboration between dentists and general practitioners in primary care clusters in Hungary
Source: Front Med (Lausanne). 2026 Jul 28;13:1879525. doi: 10.3389/fmed.2026.1879525 (PMC13456997; doi:10.3389/fmed.2026.1879525)
Supplement: Supplementary file 2 [file Table_2.docx]

Appendix 2. Interview Guide. Collaboration between dentists and general practitioners in primary care clusters in Hungary

The interview guide has been translated from Hungarian.

**Introduction of the researcher**

**Brief information about the study**

You have been invited to participate in an online interview for the study on the collaboration between dentists and general practitioners in primary care clusters in Hungary. An independent researcher from Semmelweis University will conduct the interview.

**Main purpose of the research**

The aim of the interview is to obtain a comprehensive understanding of:

- the experiences related to the establishment of primary care GP clusters;
- the areas and experiences of professional collaboration between GP and dental practices;
- the opportunities for developing interprofessional collaboration.

**Technical information**

Participation in the interview is entirely voluntary. You are under no obligation to answer all questions or to participate in the interview. Your decision to not answer a question or not to participate will be fully respected and will not result in any form of discrimination.

The interview, which will take 45-60 minutes, will be conducted with utmost confidentiality. The entire interview will be audio-recorded, but no one will be identified.

**Interview questions**

**Experiences Related to the Establishment of Primary Care GP clusters**

What were the main reasons behind your decision to join the GP cluster?

What are your general experiences regarding the operation of the GP cluster?

Does the GP cluster include only GP / dental practices, or are both professions represented?

**Areas and Experiences of Professional Collaboration Between GP and Dental Practices**

Do you know the primary care dentist/GP working in your service area? If yes, what kind of relationship or collaboration exists between you?

During patient care, for what reasons does a patient usually consult their dentist / GP, and how do you establish contact with the dentist / GP?

For which patients / problems do you usually recommend that they visit their GP / dentist?

Which diseases could a dentist / GP detect at an early stage and refer the patient to your practice?

What experiences have you had regarding collaboration with dentists / GPs within your practice / GP cluster?

Do you usually consult with each other? If yes, how regularly? How are these consultations conducted?

**Opportunities for Developing Interprofessional Collaboration**

Which factors are the most important for successful professional collaboration between dentists and GPs?

What are the greatest challenges to professional collaboration between dentists and GPs?

In your opinion, what could an ideal future professional collaboration between dentists and GPs look like?

What professional and societal benefits could result from strengthening collaboration between GP and dental practices?

Which other primary care or possibly specialist care professionals should be involved in GP clusters or in the collaboration between dental and GP practices?

**Concluding the interview**

- Are there any thoughts that have not been expressed but that you think are important to share on this topic?
